# Impacts of the COVID-19 Pandemic on Children's Sugary Drink Consumption: A Qualitative Study

**DOI:** 10.3389/fnut.2022.860259

**Published:** 2022-03-16

**Authors:** Allison C. Sylvetsky, Jasmine H. Kaidbey, Kacey Ferguson, Amanda J. Visek, Jennifer Sacheck

**Affiliations:** Department of Exercise and Nutrition Sciences, Milken Institute School of Public Health, The George Washington University, Washington, DC, United States

**Keywords:** sugar-sweetened beverages, coronavirus, diet, youth, obesity, soda, nutrition

## Abstract

The coronavirus (COVID-19) pandemic has caused striking alterations to daily life, with important impacts on children's health. Spending more time at home and out of school due to COVID-19 related closures may exacerbate obesogenic behaviors among children, including consumption of sugary drinks (SDs). This qualitative study aimed to investigate effects of the pandemic on children's SD consumption and related dietary behaviors. Children 8–14 years old and their parent (*n* = 19 dyads) participated in an in-depth qualitative interview. Interviews were recorded, transcribed verbatim, and independently coded by two coders, after which, emergent themes and subthemes were identified and representative quotations selected. Although increases in children's SD and snack intake were almost unanimously reported by both children and their parents, increased frequency of cooking at home and preparation of healthier meals were also described. Key reasons for children's higher SD and snack intake were having unlimited access to SDs and snacks and experiencing boredom while at home. Parents also explained that the pandemic impacted their oversight of the child's SD intake, as many parents described loosening prior restrictions on their child's SD intake and/or allowing their child more autonomy to make their own dietary choices during the pandemic. These results call attention to concerning increases in children's SD and snack intake during the COVID-19 pandemic. Intervention strategies to improve the home food environment, including reducing the availability of SDs and energy-dense snacks and providing education on non-food related coping strategies are needed.

## Introduction

The COVID-19 pandemic has significantly impacted the daily lives of families in the United States (U.S.) and worldwide. Public health guidance to stay at home and practice social distancing has had marked impacts on children's weight (1) and has likely influenced children's diet-related behaviors (2). Although preparation of meals at home is typically associated with lower intakes of nutrients of concern including salt, saturated fat, and added sugar (3, 4), findings of studies examining impacts of COVID-19 related stay-at-home orders on dietary intake among adults are mixed (5), and evidence on pandemic-related dietary changes among children in the U.S. is lacking.

Recent survey data in the U.S. indicate that about half of U.S. adults reported consuming more “unhealthy snacks/desserts” and approximately one-third of U.S. adults reported drinking more SDs, during, compared to before, the pandemic (6). Given that excess added sugar intake is a well-established risk factor for obesity and cardiometabolic disease (7) and children's SD intake already exceeded recommendations prior to the pandemic (8), these trends are of particular public health concern. Recent studies have reported increases in SD intake during the pandemic among U.S. adults (6, 9), yet studies examining changes in children's SD intake during the pandemic, to our knowledge, have not been conducted.

Elucidating impacts of the COVID-19 pandemic on children's SD consumption is paramount because alterations in dietary behaviors during the pandemic may persist longer-term. Furthermore, time away from school and structured activities is known to exacerbate key risk factors for overweight and obesity among children (10, 11). For example, poorer dietary intake, including higher consumption of SDs and highly processed snack foods and desserts (“junk food”), is reported during the summer months, along with greater sedentary time and use of screens (e.g., television, video games, computers) (11). These patterns of obesity risk behaviors may be worsened in the context of COVID-19 related closures and stay-at-home orders, given limited access to fresh groceries, and cancellation of youth sports and other structured programming (2). In addition, the home environment (e.g., availability of SDs at home, parental modeling of SD consumption) is a well-established contributor to excess SD consumption among children (12–14), and may be especially problematic in light of increased time spent at home during the COVID-19 pandemic. Herein, we report findings of a qualitative study designed to examine effects of the COVID-19 pandemic on children's SD consumption and related dietary behaviors.

## Materials and Methods

In-depth qualitative interviews were conducted with 19 children and their parent/guardian (hereafter parent). The children who participated were enrolled in a larger, entirely virtual, intervention study (“Stop the Pop”) designed to investigate children's physical and emotional feelings during three days of SD cessation, findings of which will be published separately. Children 8–14 years old and their parent were recruited from across the continental U.S. to participate in “Stop the Pop” using social media, community organization listservs, and parent-targeted study advertisements created by a professional recruitment agency. Interested parents completed a brief survey (administered via Qualtrics™) to determine study eligibility. Inclusion criteria were parent report that their child: (1) was between the ages of 8 and 14 years old, and (2) consumed ≥12 ounces of SDs (including regular soda, fruit drinks, fruit juice, sports drinks, and sweet tea) per day. Recruitment for “Stop the Pop” took place from November 2020 to June 2021, and the subset of 19 parent-child dyads who participated in the qualitative interviews was recruited between March 2021 and June 2021.

After providing informed consent (parent) and assent (child), and after completing the 3-day “Stop the Pop” protocol, children and their parent were invited to participate in an in-depth qualitative interview, conducted virtually via Zoom™. Interviews were conducted by a trained interviewer (ACS) using a semi-structured guide (Supplementary Material), which included questions about how the COVID-19 pandemic impacted the child's SD intake and eating behaviors, and if parental oversight of the child's SD intake had changed during the pandemic. Given that conceptualizing and articulating changes in dietary behavior during the pandemic may be cognitively challenging for children, the child and parent were interviewed together. Questions about changes in SD intake and overall diet during the pandemic were first directed to the child and then asked of the parent, whereas questions about changes in parental oversight of the child's SD intake were directed only toward parents. Data collection continued until saturation was reached, at which point, interviews had been conducted with 19 dyads. All interviews were recorded using Zoom™ and transcribed verbatim. Each dyad received a $25 Amazon gift card at the end of the interview as compensation for study participation.

Descriptive statistics were used to summarize the demographic characteristics of the child participants. Two coders (ACS and JHK) independently coded a subset (*n* = 3) of the transcripts using Microsoft Word and developed a shared codebook. Both coders then independently coded all transcripts in accordance with the shared codebook, using the NVivo Pro Software Package (version 12; QSR International Inc.; Burlington, MA, USA), and added new codes as they emerged. Once the codebook was finalized, transcripts were reviewed independently by both coders, and any discrepancies in coding were discussed. After completion of coding, the two coders independently identified key overarching themes and subthemes. Themes and subthemes were then collaboratively refined by the two coders, after which, representative quotations were selected.

## Results

Demographic characteristics of the 19 children who participated in qualitative interviews are shown in Table 1. Given that the study was designed to investigate changes in the children's SD intake and dietary behaviors during the pandemic, no demographic data were collected from parents. The sample of children was 57% female, and 63% of participants self-identified as non-Hispanic white. Forty-two percent of the participants indicated eligibility for free/reduced price lunch, and most of the children (79%) reported attending school virtually at the time of the interview.

**Table 1:** Characteristics of child participants^a^.

***N***	**19**
Age, years (mean ± SD)	11.5 ± 2.2
Female (*N*, %)	11 (57.9)
Race (*N*, %)	
White	12 (63.2)
Black	5 (26.3)
More than one race	2 (10.5)
Hispanic ethnicity (*N*, %)	2 (10.5)
Eligible for free or reduced-price lunch (*N*, %)	8 (42.1)
Attending school remotely (*N*, %)	15 (78.9)

a *No data on the demographic characteristics of the parents were collected*.

Two overarching themes emerged from the qualitative interviews. A key theme described by both children and parents was that changes in children's daily routines during the COVID-19 pandemic impacted their SD, snack, and meal intake (Table 2). The second overarching theme, as explained by parents, was that the pandemic altered parents' oversight of children's SD and snack consumption (Table 3). In addition, a minor theme identified was that changes in grocery shopping behaviors during the pandemic (e.g., stockpiling shelf stable foods due to grocery shortages, purchasing more SDs and snacks due to the whole family being at home) further promoted children's SD and snack intake.

**Table 2:** Changes in children's daily routines during the pandemic impacted their sugary drink, snack, and meal intake.

**Theme**	**Selected relevant quotations^a^**
**Subtheme**	
**Theme 1: Increases in SD consumption due to being at home**	
Access to SDs	“Well, since I'm at home almost every day, I have more access to [sugary] drinks.” (C) “I've just been drinking more stuff because it's more available to me.” (C) “Because he's home everything is accessible, where he wouldn't have that at school - he wouldn't be able to just go in the fridge and get a soda.” (P) “I think it's increased for the simple fact that they're home all day instead of at school. So, at school he's drinking water from the water fountain or his water jug that he takes to school, but since he's home he can just come down and get in the refrigerator and drink whatever.” (P)
Drinking SDs due to boredom	“When there's nothing to do, I need my energy up so I don't die of boredom. So, I try to drink sugary drinks to get my energy up so I can actually look alive.” (C) “On my breaks [from virtual school], I just get bored, so I eat or drink.” (C) “I think because one, he's bored, and two, he's got nothing else to do. It's either drink or eat something.” (P) “I think it's increased because he's at home all the time, so he's not occupied with going to class. And between classes, he doesn't have his friends here, nothing to occupy him beyond TikTok.” (P)
**Theme 2: Increases in snack intake due to being at home**	
Access to snacks	“I've been eating a lot more, because there's a lot more food to eat…I'm around food more, instead of being somewhere else for 7 hours a day.” (C) “We have a big pantry that's all stocked, and so she just has access to anything all the time.” (P) “Because he's home everything is accessible, where he wouldn't have that at school. He wouldn't be able to just go in the fridge and get a soda or get snacks.” (P) “What increases is the snacking in between because you have complete access to your kitchen all day whereas if you were at school you would not.” (P)
Lack of scheduled eating times	“Because he's not at school, so he's here able to get a snack or come down and make something whenever he feels like it.” (P) “I think our kids have all turned into grazers and eat whenever they feel hungry, which definitely was not the norm during regular school because they were only allowed at snack time and lunchtime. But at home they've been allowed a little bit more flexibility.” (P) “The accessibility to snacks is different, because you taking your lunch box is one thing, and then, you know, given your time constraints at school, you can't just have a snack whenever you want it.” (P) “After being in school all day, she will eat whatever's there because she's hungry because she doesn't really eat the school lunches; so, she's extremely hungry when she gets home…Now that she's home, the food is here, and she doesn't really want to eat, and she just wants to snack here and there.” (P)
Snacking due to boredom	“Before, I always had something to do. But now I'm just like...nothing to do. You come from school and nothing else. That's it. End of the day. Snack, snack, snack.” (C) “I think I eat a lot more often [during the pandemic] because I sat at home and did absolutely nothing, so I just ate.” (C) “Just definitely more snacking because there is more just sitting around, playing games on the computer. It's just us around the house…everyone's just in shorts and t-shirt and just grabbing a package of goldfish or something, in the middle of class.” (P) “Definitely a lot more snacking... not necessarily out of hunger, but boredom.” (P)
**Theme 3: Healthier choices due to being home**	
Being “on the go” less frequently	“We have less convenience foods...before the pandemic, our kids were involved in scouts and 4H and we were running a lot more. So, we were grabbing, you know quickie stuff.” (P) “I'm not buying them [snack foods] as often because I don't feel that we need the ‘on the go' things so much, because we're home.” (P) “I can cut up an apple, we're not in the car, or on the road, so we don't have to have the easy, open snacks anymore.” (P)
Not going to school or activities	“On the way home [from school], I'd just go buy soda for myself. But, but when I was in quarantine like I didn't do any of that.” (C) “When I was in middle school, I would eat out and I would go outside for lunch every single day and get a soda every single day.” (C) “Even if the school is open, they have nothing going on there… no events, no parties, no celebrations, no birthdays [with SDs]; nothing is going on either in school or outside of school.” (P)
**Theme 4: Changes in daily routines impacted meal preparation and intake**	
More child involvement in cooking	“She's also looking online for more stuff…mainly recipes, she started cooking on her own.” (P) “We're actually cooking. We're cooking together every night now.” (P) “Now that he's helping cook, he's actually putting onions in things and doesn't mind them.” (P)
Cooking instead of eating out	“We didn't do takeout at all for like the first like 7-8 months, so like we were cooking at home a lot, everyone was eating like fresh, you know, like homemade meals.” (P) “I cook more. So, we're eating more healthier meals, because I'm cooking every day, whereas before it was “go-go-go” and I wasn't always cooking. It was more, ‘let's grab a bowl of cereal'– but at home, I'm cooking more.” (P) “She's been here, so rather than just taking a sack lunch to school, it just allows more time to be able to come down and make different foods…So, she's been eating more variety, healthy foods. Yeah making good choices.” (P)
Skipping breakfast	“I'll stay in bed and like, usually there's times where I'll just stay in bed and I won't eat until someone actually makes me eat.” (C) “I used to eat breakfast before school, but now I don't really eat breakfast that much.” (C) “I definitely noticed that she hasn't been eating breakfast. I work from home quite a bit lately as well and sometimes I don't even see her until lunch time.” (P) “In school, there would be breakfast and lunch...at home, he won't eat breakfast.” (P)
**Theme 5: Changes in daily routines impacted the types of beverages consumed**	
Consuming different types of beverages	“I usually have a juice box every day because I take it for lunch, but I didn't need to drink a juice box every day [during the pandemic], so I usually drink something else.” (C) “When he had his sports and stuff, it was more the sport drinks, instead of everything else. So, I guess the volume would have been similar, but what it is he's drinking has changed.” (P) “I think more or less, a better way to explain is not that her sugary consumption has increased, so much as what particular thing if that makes more sense.” (P)

a *Child responses are indicated by (C) and parent responses by (P) following the quotation*.

**Table 3:** The pandemic altered parents' oversight of children's SD consumption.

**Theme**	**Selected relevant quotations^a^**
**Subtheme**	
**Theme 1: Less restrictions on children's SD consumption**	
Provision of SDs as a coping mechanism	“As ridiculous and counterproductive as it sounds, I think that we were a lot more lenient. I've noticed I was buying a lot more treats and stuff…foods that the kids would be excited about which is usually sugary stuff. We went a little wild with the treats…just trying to compensate for them being stuck at home and bored.” (P) “That was the only way I could get him to sit down for some of his classes…was to give him his fruit juice or whatever he wanted.” (P) “I'm a little more lenient. You tend to, you know, feel bad for situations.” (P)
**Theme 2: More child autonomy in making beverage choices**	
Less parental oversight	“Before COVID, my mom used to be strict-strict about drinks, I wouldn't necessarily be drinking things besides water, because, instead of now where you have to get at least one drink of water a day, before you could only have one juice a day.” (C) “He doesn't ask anymore. When he goes to grab Capri Suns, he used to ask. Now he just grabs it, and doesn't say anything. I've caught him sitting playing video games, with the whole Capri Sun box next to him.” (P) “Previous to the pandemic, I was a little more mindful of what he was drinking. But now we're 400 days in of being together all the time. And I guess we've gotten to a point where if you're thirsty, just get something to drink. Just grab a drink.” (P)
**Theme 3: More parental awareness of child's SD intake**	
More parent awareness	“I think we actually see more of their consumption, because at school, we didn't see what she was drinking other than what we either included in cold lunch, or we knew she was getting milk at snack and lunch time.” (P) “[Before the pandemic] I didn't know what he drinks because he gets money and never tells us what he's drinking, and now, it's more in control. So, we will see what he's drinking.” (P) “It [the pandemic] made me realize how much sugary drinks we have in this house and how much is being consumed.” (P)

a *Child responses are indicated by (C) and parent responses by (P) following the quotation*.

### Changes in Children's Daily Routines During the COVID-19 Pandemic Impacted Their SD, Snack, and Meal Intake

As shown in Table 2, five key themes related to how changes in children's daily routines during the pandemic impacted their SD and snack intake were identified. Most notably, increased time spent at home, rather than in school, promoted excess consumption of SDs and snacks among children, according to both children and their parents. Increased SD and snack intake at home was commonly attributed to having unrestricted access to SDs and snacks, the child experiencing boredom, and a lack of scheduled or structured eating times. Skipping breakfast when attending school virtually was also commonly reported by children and corroborated by parents. However, parents also explained that changes in the child's daily routine during the pandemic led to favorable dietary changes, including making healthier choices as a result of not being “on the go” and cooking more meals at home, as opposed to eating out. Some children and parents also described a shift in the types, rather than the volume, of SDs the child consumed as a result of the pandemic; for instance, consuming fewer juice boxes and sports drinks, due to not needing to bring a lunch to school and having fewer sports and extracurricular activities.

### The COVID-19 Pandemic Altered Parents' Oversight and Views of Children's SD and Snack Consumption

As shown in Table 3, three key themes were identified pertaining to changes in parental oversight of the child's SD intake during the pandemic. Parents described removing prior restrictions on SDs, and in some cases, providing their children with SDs as a means of helping them cope with disturbances to daily life caused by the pandemic. For example, parents reported providing their child with SDs as a treat to make the child happy, and being more lenient about allowing their child to have SDs due to feeling bad for their child during the pandemic. Parents also described allowing their child more autonomy in making their own beverage choices during the pandemic. For instance, some parents explained that prior expectations that the child ask before helping themselves to SDs were no longer applicable. While these changes in parental oversight of their child's SD intake were commonly described as facilitators of increased SD consumption during the pandemic, some parents reported that being home together made them more aware of their children's SD consumption and/or made it easier for them to restrict their children's SD intake during the pandemic.

## Discussion

Our findings demonstrate that spending more time at home and out of school during the COVID-19 pandemic resulted in perceived increases in children's SD and snack intake. These findings are consistent with several recent studies reporting unfavorable effects of the COVID-19 pandemic on dietary intake among adults (6, 9), as well as recent reports of unhealthful dietary changes among children in other countries, including Italy (15) and China (16). Increases in SD intake and snacking during the pandemic are also supported by a large body of evidence demonstrating that unhealthy weight gain among children occurs disproportionately when out of school (i.e., during the summer months), compared with during the school year (17, 18).

Greater access to SDs and snacks while at home was described by both parents and children as the predominant contributor to reported increases in children's SD and snack consumption during the pandemic. This is not surprising, as the contribution of physical aspects (e.g., availability) and social aspects (e.g., parental modeling, family meal practices) of the home environment to children's dietary intake is well-established (12, 14). Availability of SDs in the home is positively associated with SD intake among youth (19, 20), and similar findings have been reported with regard to intake of energy-dense snacks (21). A recent cross-sectional study in the U.S. indicated that one-third of parents increased the amount of high-calorie snack foods, desserts, and sweets available in the home during the pandemic, while nearly half (47%) reported increases in the availability of non-perishable processed foods (22). These shifts in the home food environment during the COVID-19 pandemic may have further exacerbated increases in children's SD and snack intake behaviors. In addition, parent modeling of SD intake is another well-described contributor to children's SD intake (23). Given that SD intake also increased among adults during the COVID-19 pandemic (6), amplified parent modeling of SD consumption may have further contributed to the reported increases in children's SD intake.

Children and parents in our study also commonly described the lack of scheduled meal and snack times, and cancellation of extracurricular activities, as reasons for reported increases in their children's SD and snack intake. This finding is consistent with the “structured days hypothesis (SDH)” (10), which has been proposed to explain accelerated weight gain among children during the summer months. The SDH posits that compared with the school year, during which children follow a consistent, structured, and regimented schedule with adult supervision, the summer months typically consist of less structure and more child autonomy (10). This lack of structure provides children with more opportunities to eat (as opposed to scheduled snack and mealtimes in school) and may increase the likelihood that children make poor dietary choices (10).

Parents also described loosening restrictions on their child's dietary intake during the pandemic, which has also been reported among parents of younger children (24), and providing SDs and treats to help their children cope with disruptions to daily life. These behaviors are concerning because indulgent parenting (25), where children have freedom to eat and drink whatever they wish, and emotional eating (26), where intake of foods high in sugar and/or fat to reduce the intensity of negative emotions, are both associated with excess weight gain among children (25, 26). Marked increases in depression and anxiety among children during the COVID-19 pandemic (27) may also have contributed to reported increases in SD and snack intake, given that psychological distress is associated with overeating among youth (28).

Despite the nearly unanimously reported increases in children's SD intake and snacking, parents reported some favorable impacts of the COVID-19 pandemic on children's diets, specifically with regard to cooking at home and eating healthier meals. Increases in cooking during the pandemic were reported in a recent scoping review (5), which also demonstrated that the pandemic had both favorable and unfavorable effects on dietary intake. Parents in our study explained that having more time and having fewer other commitments (i.e., not being on the go) were key reasons for cooking more frequently during the pandemic, consistent with prior work describing a perceived lack of time as a barrier to cooking healthy meals at home (29, 30). As has been reported in other recent publications (31, 32), parents also explained that their child was more involved in cooking meals during the pandemic. Given that cooking at home is associated with healthier dietary patterns (33), the shift toward more cooking during the pandemic may lay the groundwork for sustained improvements in meal healthfulness beyond the pandemic. Greater child involvement in cooking also holds promise, as learning cooking skills at an early age is positively associated with higher diet quality (34). However, it is unclear whether these benefits will persist, given that by mid-2021, national food sales outside the home began to exceed food at home for the first time since the pandemic began (35).

While our findings offer novel insights into impacts of the COVID-19 pandemic on children's SD, snack, and meal consumption, the study was subject to several limitations. First, the children's responses may have been influenced by interviewing the parent and child together, leading to possible contamination of the data collected. In addition, the small sample size precluded comparing differences in pandemic-related dietary changes based on participants' race, ethnicity, or household income. This is an important limitation because youth from low-income and/or minority backgrounds are most susceptible to weight gain when out of school (36); thus, increases in SD and snack intake reported during the pandemic may worsen already marked health disparities. Another limitation was the enrollment of children who reported habitual daily consumption of SDs (per inclusion criteria for “Stop the Pop”); therefore, the extent to which the pandemic may have impacted SD intake among less frequent SD consumers could not be assessed. The parents' work environment (remote vs. in-person) also may have changed as a result of the pandemic and influenced children's SD intake and related dietary behaviors; however, data on the parent's work environment were not collected. It is also important to note that participants in the present qualitative study comprised a subset of individuals participating in a larger intervention study of short-term SD cessation. It is therefore possible that these individuals may have already had a high awareness or concern about SD intake, and thus, their description of changes in SD intake behaviors during the pandemic may not reflect those of the general population.

Taken together, our findings call attention to concerning increases in SD and snack intake among children during the COVID-19 pandemic, the effects of which may be partially offset by increases in cooking and consumption of healthier meals. Surveillance of children's diets throughout and following the pandemic is needed, as the extent to which the perceived increases in SD and snack consumption will persist longer-term is presently unclear. While these dietary changes were reported in the unique context of the COVID-19 pandemic, our findings may apply more broadly to other prolonged periods of unstructured, out-of-school time (i.e., the summer recess). Intervention strategies to improve the home food environment, such as reducing the availability of SDs and energy-dense snacks are needed, along with efforts to educate parents about optimal food parenting practices and equip children with more adaptive, non-food related coping skills.

## Data Availability Statement

The raw data supporting the conclusions of this article will be made available by the authors, without undue reservation.

## Ethics Statement

The studies involving human participants were reviewed and approved by Institutional Review Board at the George Washington University. Written informed consent to participate in this study was provided by the participants' legal guardian/next of kin.

## Author Contributions

AS, JK, AV, and JS designed the research. AS and JK performed the analyses. AS wrote the first draft of the manuscript. All authors were involved in editing the manuscript and approved the final version.

## Funding

This project was supported by a KL2 Career Development Award (PI: AS), under Parent Award numbers UL1TR001876 and KL2TR001877 from the National Institutes of Health (NIH) National Center for Advancing Translational Sciences (NCATS).

## Author Disclaimer

The contents are solely the responsibility of the authors and do not necessarily represent the official views of the NIH or NCATS.

## Conflict of Interest

The authors declare that the research was conducted in the absence of any commercial or financial relationships that could be construed as a potential conflict of interest.

## Publisher's Note

All claims expressed in this article are solely those of the authors and do not necessarily represent those of their affiliated organizations, or those of the publisher, the editors and the reviewers. Any product that may be evaluated in this article, or claim that may be made by its manufacturer, is not guaranteed or endorsed by the publisher.
