# Supplementary material for: Impacts of the COVID-19 Pandemic on Children's Sugary Drink Consumption: A Qualitative Study
Source: Front Nutr. 2022 Mar 16;9:860259. doi: 10.3389/fnut.2022.860259 (PMC8966582; doi:10.3389/fnut.2022.860259)
Supplement: Supplementary file 1 [file Data_Sheet_1.pdf]

Qualitative Interview Guide

1. **How do you [child] think that the COVID-19 pandemic has impacted your sugary drink intake?**

    - Would you say that your sugary drink intake has increased or decreased during the pandemic?
    - Why?

2. **How do you [parent] think that the COVID-19 pandemic has impacted your child's sugary drink intake?**

    - Would you say that your child's sugary drink intake has increased or decreased during the pandemic?
    - Why?

3. **How much oversight do you have over [insert child's name] beverage intake? Has this changed during the COVID-19 pandemic?**

    a. If yes, how so?
    b. Would you say that you have more or less oversight over your child's sugary drink intake?
    c. Is it easier to more difficult to monitor their beverage intake during the pandemic?

4. **How about for your [ask child]/their [ask parent] other dietary behaviors? Have you noticed any differences in the context of COVID-19?**

    a. Has the preparation of meals changed?
    b. Has the healthfulness of meals changed?
    c. Have you noticed changes in your/your child's snacking behaviors?

5. **Is there anything else that either of you would like to add about how COVID has impacted your/your child's eating and drinking behaviors or your experience in the study?**
